# Single-molecule magnet behavior in 2,2’-bipyrimidine-bridged dilanthanide complexes

**DOI:** 10.3762/bjnano.7.15

**Published:** 2016-01-28

**Authors:** Wen Yu, Frank Schramm, Eufemio Moreno Pineda, Yanhua Lan, Olaf Fuhr, Jinjie Chen, Hironari Isshiki, Wolfgang Wernsdorfer, Wulf Wulfhekel, Mario Ruben

**Affiliations:** 1 Institute of Nanotechnology (INT), Karlsruhe Institute of Technology (KIT), Hermann-von-Helmholtz-Platz 1, D-76344 Eggenstein-Leopoldshafen, Germany; 2 Université Grenoble Alpes / CNRS, Institut NEEL, 25 rue des Martyrs, F-38000 Grenoble, France; 3 Physikalisches Institut (PI), Karlsruhe Institute of Technology (KIT), Wolfgang-Gaede-Str. 1, D-76131 Karlsruhe, Germany,; 4 Institut de Physique et Chimie des Matériaux de Strasbourg (IPCMS), CNRS-Université de Strasbourg, 23 rue du Loess, BP 43, F-67034 Strasbourg Cedex 2, France

**Keywords:** bipyrimidine, diketone, hysteresis, lanthanide, magnetism

## Abstract

A series of 2,2’-bipyrimidine-bridged dinuclear lanthanide complexes with the general formula [Ln(tmhd)_3_]_2_bpm (tmhd = 2,2,6,6-tetramethyl-3,5-heptanedionate, bpm = 2,2’-bipyrimidine, Ln = Gd(III), **1**; Tb(III), **2**; Dy(III), **3**; Ho(III), **4** and Er(III), **5**) has been synthesized and characterized. Sublimation of [Tb(tmhd)_3_]_2_bpm onto a Au(111) surface leads to the formation of a homogeneous film with hexagonal pattern, which was studied by scanning tunneling microscopy (STM). The bulk magnetic properties of all complexes have been studied comprehensively. The dynamic magnetic behavior of the Dy(III) and Er(III) compounds clearly exhibits single molecule magnet (SMM) characteristics with an energy barrier of 97 and 25 K, respectively. Moreover, micro-SQUID measurements on single crystals confirm their SMM behavior with the presence of hysteresis loops.

## Introduction

The application of magnetic molecular compounds within molecular electronic devices is combined in the progressive field of spintronics. An anisotropic spin is confined by the ligand field of the metal complex, which is typically of a few nm in dimensions. It is proposed that the ultimate size limits of modern electronics and information processing can be tackled by such device geometry [1–3].

Research on compounds with single-molecule magnet (SMM) characteristics discovered striking advantages that make this class of molecules promising candidates for molecular spintronics [4]: (a) Each molecule behaves as a single spin domain, thus they could act as the base of a high-density information storage or processing unit; (b) They possess an energy barrier to inversion of total spin, leading to slow magnetic relaxation and magnetic hysteresis at low temperatures. Combined with their long coherence times they could open the door to quantum computing [5–6].

After the first SMM was discovered in 1980 [7–8], for the next 15 years the SMM field was dominated by cluster compounds containing mainly high-spin Mn(III). In such compounds, the SMM behavior was due to a combination of the Jahn–Teller distortion of the Mn(III), the ferromagnetic alignment of the Mn(IV), and overall antiferromagnetic exchange between the Mn(III) and Mn(IV) leading to a *S* = 10 ground state [9]. To date, the largest anisotropy barrier observed in a transition metal SMM is 226 cm^−1^ in the Fe(II) complex [Fe(C(SiMe_3_)_3_)_2_]^−^ [10]. Recently, molecular compounds employing lanthanide ions led to a considerable increase in strength of molecular magnets due to the observation of the SMM character at the single-ion level [11–12].

SMMs based on 4f ions possess larger thermal energy barriers for magnetization reversal caused by their large single-ion magnetic anisotropy, which originates from spin–orbit coupling and crystal-field splitting of the 4f ions. Technological and structural development of lanthanide SMM compounds to access specific surface deposition drive the innovation towards device applications [13–16].

The design of a suitable system that includes quantum bits (qubits) and quantum gates (qugates) is the main challenge to realize quantum computing. There, the electronic spins of magnetically anisotropic lanthanide ions can possibly act as basic units of quantum computing, i.e., as qubits. Universal qugates may be engineered by designing one molecule with two interacting lanthanide ions [17]. We are particularly interested in generating dinuclear lanthanide complexes with a bridging ligand that provides a communication pathway between the lanthanide ions. The 2,2’-bipyrimidine (bpm) ligand has been selected for many transition metal complexes to bridge metal ions and it acts as a connector to influence emission and magnetic properties [18–21]. An adsorption-site-dependent zero-bias (Kondo) resonance was clearly observed in the dinuclear transition metal complexes with the bpm bridging ligand [20]. In the same molecular architecture, the substitution of Ising-like spins, such as lanthanides, can offer a weak exchange coupling to study their possible implementation as qugates. Dinuclear lanthanide complexes with bpm as the bridge were reported before, but only a few studies describe their magnetic properties [22–30]. In this work we report the synthesis, characterization and single-crystal structure determination of five examples of homo-dinuclear complexes of tris-β-diketonate adducts of Gd(III), Tb(III) [30], Dy(III), Ho(III) and Er(III) with a bpm bridging ligand. We employed 2,2’,6,6’-tetramethyl-2,4-heptanedionate (tmhd) ligands as peripheric ligands, providing overall charge-neutral compounds. The magnetic behavior of the compounds was measured using AC, DC and micro-SQUID magnetometry techniques. The homo-dinuclear complexes of Dy(III) and Er(III) show single-molecule magnet behavior featuring hysteresis loops. The [Tb(tmhd)_3_]_2_bpm was sublimated on Au(111) surfaces and scanning tunneling microscopy results are presented in this report.

## Experimental

### Synthesis

Solvents and reagents were of commercial grade and used without further purification. **1**–**5** were prepared by modification of published procedures [29]. To a mixture of Ln(tmhd)_3_ [Ln = Gd(III) **1**; Tb(III) **2**; Dy(III) **3**; Ho(III) **4** and Er(III) **5**] (1 mmol) and bpm (0.5 mmol) in a 50 mL flask absolute ethanol (20 mL) was added. The suspension was stirred overnight at room temperature, leading to a precipitate that was then collected by filtration. The powder was washed with *≈*10 mL of cold absolute ethanol and dried at 90 °C overnight. The powder was dissolved in a minimum volume of CHCl_3_ at room temperature, immediately filtered to remove insoluble particles, and then reprecipitated by the addition of cold absolute ethanol. The precipitate was again filtered and dried at 90 °C. X-ray diffraction quality crystals were formed by recrystallization from Et_2_O for **2** and **3** at −17 °C and by layering EtOH onto a CHCl_3_ solution of **1**, **4** and **5** at room temperature, respectively. Results of elemental analyses and isolated yields are given in Table 1.

**Table 1:** Elemental analysis and yield (%) for compounds **1**–**5**.

	Formula	Yield^a^	Elemental analysis: found (calculated)		
			C	H	N

**1**	[Gd(thmd)_3_]_2_bpm	33%	56.33 (56.53)	8.05 (7.69)	3.57 (3.56)
**2**	[Tb(thmd)_3_]_2_bpm·Et_2_O	42%	59.08 (59.06)	8.21 (8.04)	3.61 (3.72)
**3**	[Dy(thmd)_3_]_2_bpm	42%	56.22 (56.15)	8.08 (7.64)	3.57 (3.54)
**4**	[Ho(thmd)_3_]_2_bpm	43%	56.23 (55.98)	8.23 (7.62)	3.55 (3.53)
**5**	[Er(thmd)_3_]_2_bpm	43%	55.71 (55.82)	7.60 (7.60)	3.52 (3.52)

^a^Calculated based on the lanthanide starting material.

### Physical measurements and instrumentation

IR transmission measurements of pressed KBr pellets were recorded at room temperature with a Perkin Elmer Spectrum GX FT-IR system spectrophotometer. Elemental analysis data were collected on an ELEMENTAR Vario Micro Cube. NMR spectra were carried out on a Bruker Ultrashield 500 PLUS spectrometer.

**Scanning tunneling microscopy.** The STM measurements were realized with a homebuilt scanning tunneling microscope. The sample preparation, other than molecule deposition and Ar sputtering, were carried out in ultrahigh vacuum conditions (≈10^−10^ mbar). Degassing of the [Tb(thmd)_3_]_2_bpm compound was performed carefully by heating to 373 K in a ceramic crucible for hours prior to evaporation. The Au(111) single crystal substrate was cleaned with a standard Ar sputtering and annealing process in a separate preparation chamber. After annealing and cooling down to room temperature, the substrate was transferred to a molecule deposition chamber and was exposed to a molecule flow of about 0.01 monolayer/sec for several ten second steps at a sublimation temperature of about 433 K. The pressure during deposition was ca. 2 × 10^−7^ mbar. After deposition, the samples were transferred to the STM chamber immediately and cooled down to 5 K. During the measurement, the sample temperature was kept at 5 K.

**Magnetic measurements.** Magnetic susceptibility measurements were collected using a Quantum Design MPMS^®^3 and MPMS-XL SQUID magnetometer. DC susceptibility measurements for all compounds were performed at temperatures ranging from 2 to 300 K, using an applied field of 1 kOe. The AC data were collected using an oscillating magnetic field of 3.5 Oe. All data were corrected for diamagnetic contributions from the eicosane and core diamagnetism, estimated using Pascal’s constants [31]. Low temperature (0.03–5 K) magnetization measurements were performed on single crystals using a micro-SQUID apparatus at different sweep rates between 0.280 and 0.002 T s^−1^ [32]. The applied field was parallel to the experimentally observed easy axis of magnetization.

**X-ray data collection and structure solution**. Data collection for all complexes was carried out on a STOE StadiVari 25 diffractometer with a Pilatus300 K detector using GeniX 3D HF micro focus X-ray source (Mo Kα, λ = 0.71073 Å). The structures were solved by direct methods and refined employing full-matrix least-squares refinement against *F^2^* using SHELX2014 [33] and OLEX2 [34] packages. H atoms were added at idealized positions on their respective parent atoms. Full crystallographic details can be found in CIF format (see the Cambridge Crystallographic Data Centre database, 1427566, 1419831–1419834). These data can be obtained free of charge via http://www.ccdc.cam.ac.uk/conts/retrieving.html (or from Cambridge Crystallographic Data Centre, 12 Union Road, Cambridge CB21EZ, UK; fax: (+44)1223-336-033; or deposit@ccdc.cam.ac.uk) (See Table 2).

**Table 2:** Crystallographic information for clusters **1**–**5**.

	**1**	**2**	**3**	**4**	**5**

Formula	C_75_H_121_Cl_3_Gd_2_N_4_O_12_	C_78_H_130_N_4_O_13_Tb_2_	C_78_H_130_Dy_2_N_4_O_13_	C_75_H_121_Cl_3_Ho_2_N_4_O_12_	C_74_H_122_Er_2_N_4_O_12_
fw/g mol^−1^	1691.60	1649.69	1656.85	1706.96	1594.27
Crystal system	monoclinic	triclinic	triclinic	monoclinic	triclinic
Space group	*P*2_1_	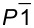	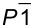	*P*2_1_	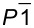
*a*/Å	10.8114(3)	10.8604(5)	10.8535(4)	10.8520(5)	10.8332(5)
*b*/Å	29.6046(10)	13.8827(6)	13.8502(6)	29.5505(10)	12.5416(6)
*c*/Å	13.8460(4)	14.8544(6)	14.8305(6)	13.7466(6)	16.8646(8)
α/°	90	92.101(3)	92.011(3)	90	106.948(4)
β/°	106.850(2)	93.253(3)	93.342(3)	106.945(4)	101.337(4)
γ/°	90	107.539(3)	107.530(3)	90	105.671(4)
V/Å^3^	4241.4(2)	2128.66(16)	2118.97(15)	4216.9(3)	2013.84(18)
*Z*	2	1	1	2	1
ρ_calcd_/g cm^−3^	1.325	1.287	1.298	1.344	1.315
*T*/K	180.15	180.15	180.15	180.15	180.15
μ/mm^−1^	1.699	1.704	1.806	2.078	2.125
R_1_(*I* > *2*σ)(*I*))^a^	0.0225	0.0175	0.0231	0.0216	0.0429
*w*R_2_^a^	0.0592	0.0432	0.0604	0.0479	0.1166

^a^R_1_ = ∑||*F*_0_| − |*F*_c_|| / ∑|*F*_0_|, *w*R_2_ = [∑*w*(*F*_0_^2^ − *F*_c_^2^)^2^ / ∑*w*(*F*_0_)^2^]^1/2^.

## Results and Discussion

### Synthesis and crystal structure

2,2'-bipyrimidine was chosen as the bridging ligand for its general chemical stability, for its strong σ-donor and π-acceptor characteristics and its symmetric shape. In combination these properties stabilize the symmetric coordination on both 1,4-diimine coordination sites. For homo-dinuclear complexes, the electronic or magnetic interaction between two metal centers remains unperturbed by asymmetry. Additionally, the coordination of the peripheral ligands tris(tetramethylheptanedionato) lanthanide(III) for f-series metal ions results in charge-neutral homo-dinuclear compounds; an aspect potentially important for later deposition of the compounds in device environments by sublimation techniques.

Compounds **1**–**5** were synthesized by adding Ln(tmhd)_3_ (Ln = Gd(III) **1**; Tb(III) **2**; Dy(III) **3**; Ho(III) **4** and Er(III) **5**) to 2,2'-bipyrimidine and stirring the mixture in EtOH overnight. The molecular structure of compounds **1**–**5** is isostructural and the complexes crystallize in two different unit cells depending on the lanthanide source. Compounds **2**, **3**, and **5** crystallize in the triclinic 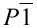 space group, while **4** crystallizes in the monoclinic *P*2_1_ space group; a behavior that can be attributed to the lanthanide contraction along the series of f-elements.

**Figure 1:**
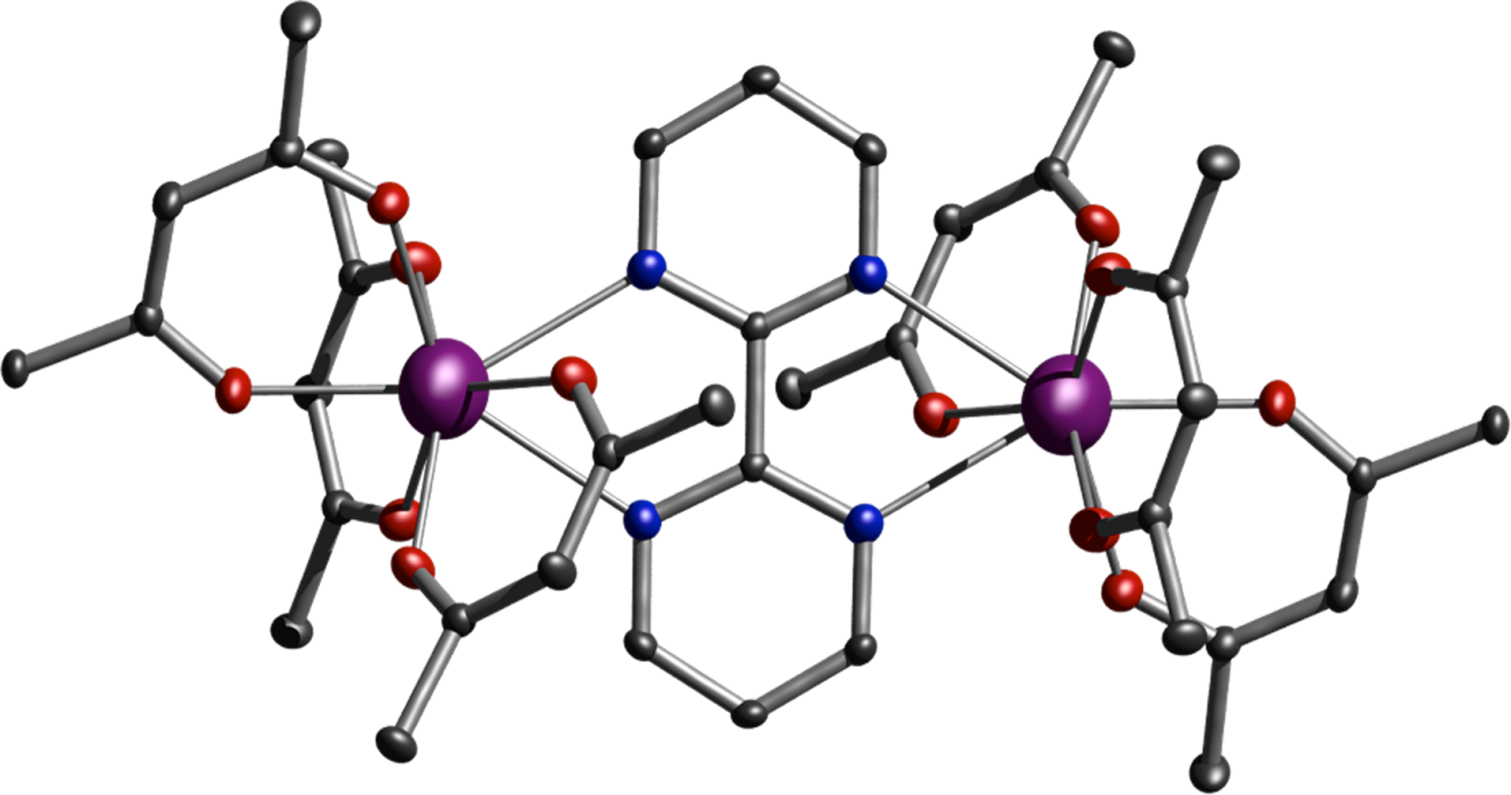
Molecular structure of complex **3** obtained from single-crystal diffraction. Dy(III) ions are marked in purple, oxygen in red, nitrogen in blue and carbon in dark grey. Hydrogen and methyl groups are omitted for clarity. Thermal ellipsoid of C, N and O (50% probability), whilst Dy is represented employing 99% probability.

The molecular structure of **3** is given here in detail as a representative case for all compounds **1**–**5** (see Figure 1). The bpm moiety of the dilanthanide complexes resides on a crystallographic inversion center, whilst the individual ions are localized between the two aromatic rings of the bpm bridging ligand. The two metal centers are equivalent by symmetry. Each metal ion is eight-coordinated by six oxygen atoms from the tmhd and two nitrogen atoms of the bpm, resulting in an O_6_N_2_ donor set. The coordination polyhedron around the lanthanide ion can be most closely described as a square antiprism (see Supporting Information File 1, Table S1) (*D*_4_*_d_*, CShM of 0.605) [35–36]. The coordination polyhedron, as obtained from the single crystal analysis, is depicted in Supporting Information File 1, Figure S1. The square planes of the polyhedron are defined by the O1, O2, N1, N2 and the O3, O4, O5, O6 atoms, respectively. The lanthanide ion sits closer to the plane that is defined by four oxygen atoms. The normal vector distances between the Ln atoms and the O3, O4, O5, O6 planes are about 1.13 Å in all five compounds **1**–**5**, while the O1, O2, N1, N2 planes vary between 1.42 Å and 1.45 Å (Supporting Information File 1, Table S1). The Ln(III) atoms are bound to the bpm ligand slightly above and below the plane defined by the aromatic pyrimidine rings of the bpm ligand, creating a chair-like conformation (Figure 1). The point group of the molecules varies between *C*_1_ and *C_i_* depending on the degree of distortion within the molecule. The two coordination spheres within each dinuclear compound are more or less inversion-symmetry-related to each other, resulting in the formation of an achiral *meso*-form of the polyhedra (see Supporting Information File 1, Figure S1).

The bond lengths between the lanthanide ions and the coordinating oxygen and nitrogen atoms of the ligands are summarized in Table 3. They decrease along the lanthanide series, with a corresponding decrease in ionic radii referred to as the lanthanide contraction. The distances between the two lanthanide ions have the same trend and are also showed in Table 3. These values are often observed in other known bpm-bridged Ln(III) dimers [28–29].

**Table 3:** Range of bond lengths between Ln and coordinating atoms, Ln···Ln distance in complexes **1**–**5**.

	Ln–O [Å]	Ln–N [Å]	Ln···Ln [Å]

**1**	2.277(3)–2.363(3)	2.613(4)–2.657(4)	6.8752(2)
**2**	2.265(2)–2.343(2)	2.599(2)–2.616(2)	6.8124(3)
**3**	2.260(2)–2.330(2)	2.581(2)–2.604(2)	6.7841(3)
**4**	2.243(6)–2.327(5)	2.583(8)–2.596(8)	6.7933(2)
**5**	2.241(4)–2.296(4)	2.578(4)–2.587(4)	6.7545(3)

### Magnetic properties

**Static susceptibility measurements.** The static magnetic behavior for all complexes was investigated on polycrystalline samples from 2 to 300 K under an applied DC magnetic field (*H*) of 1 kOe, whilst magnetization as a function of applied field was investigated in the field and temperature range of 0–7 T and 2–5 K, respectively.

**Figure 2:**
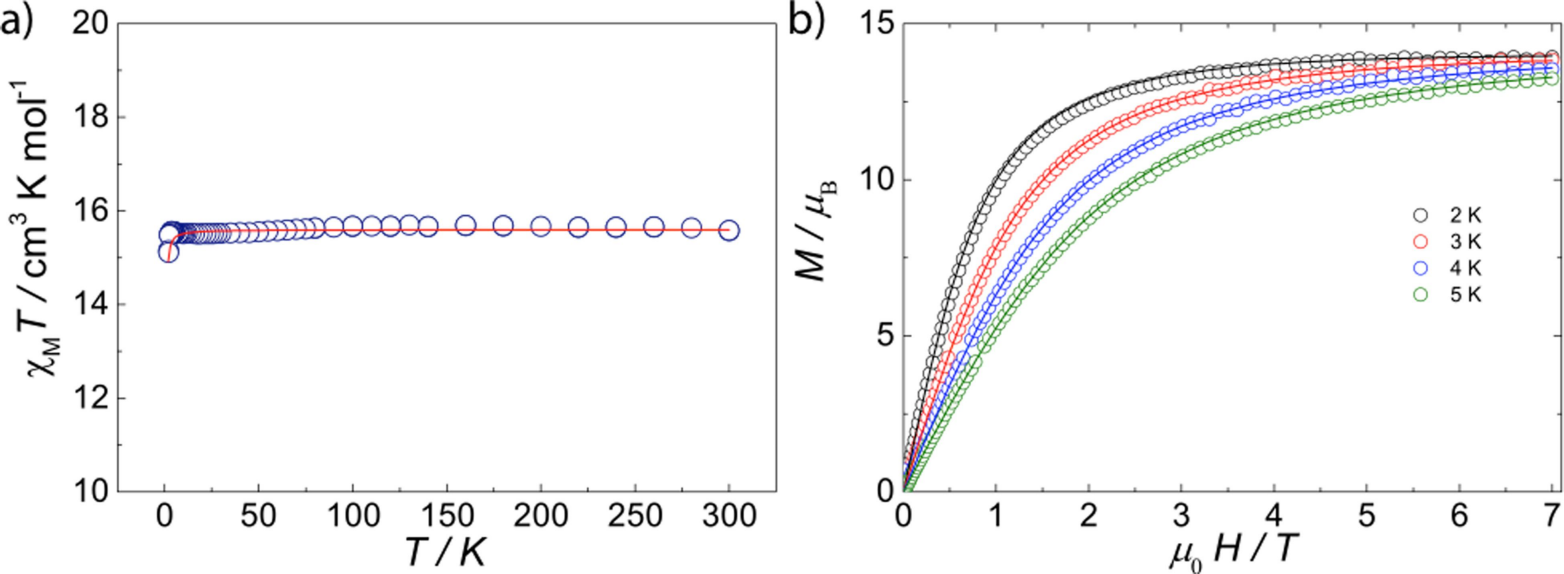
(a) Experimental χ_M_*T*(*T*) and fitting (red trace) for compound **1** (parameters for fitting are described in the text); (b) Experimental *M*(*H*,*T*) (open circles) and Brillouin functions at given temperatures employing *g* = 2.00. The *M*(*H*,*T*) can similarly be reproduced employing the small *J* used to reproduced χ_M_*T*(*T*).

**Figure 3:**
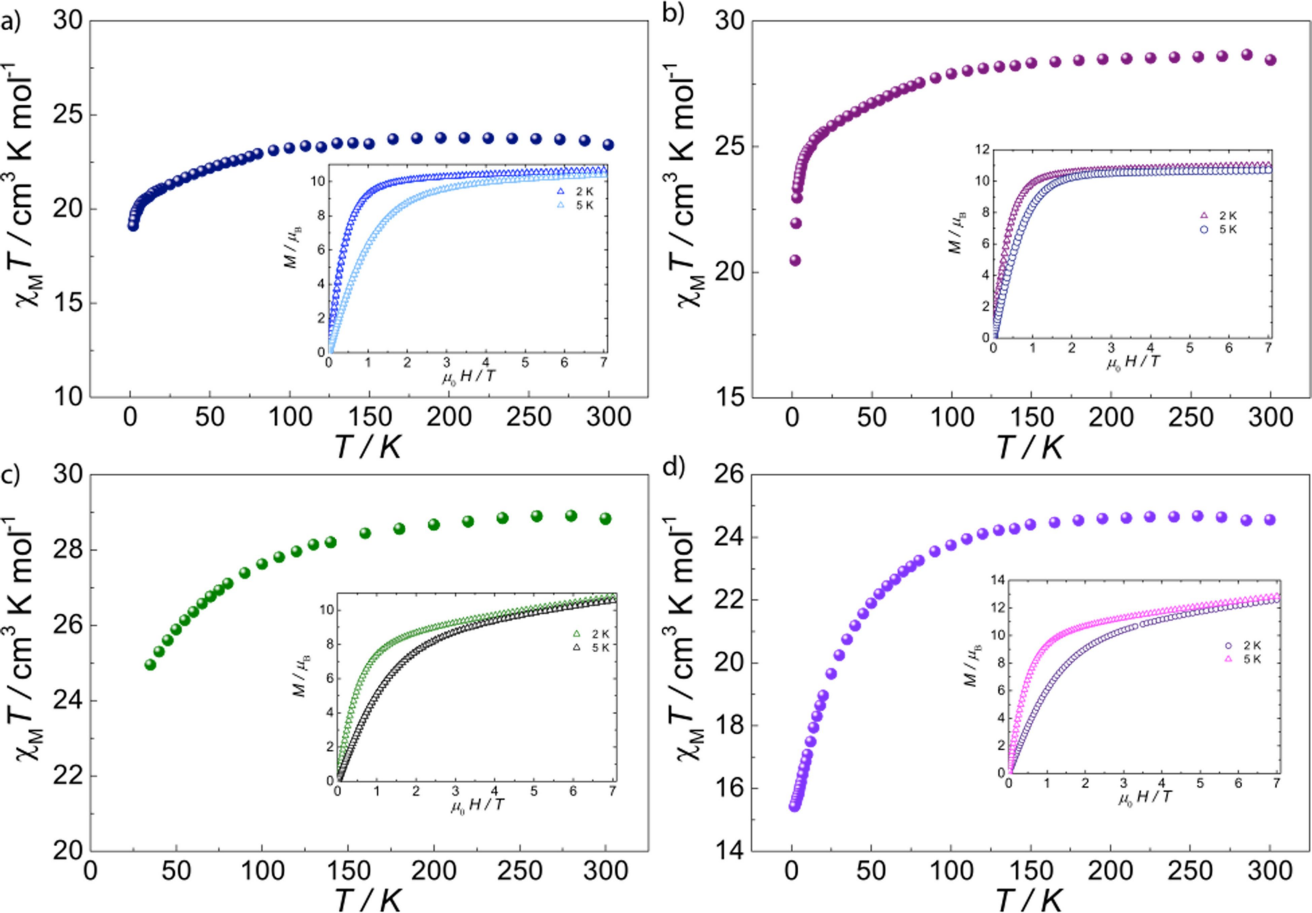
Molar magnetic susceptibility (χ_M_*T*) vs *T* plot for **2**–**5** under 0.1 T DC field and molar magnetization (*M*_β_) as a function of applied magnetic field (*H*) at 2 and 5 K (inset) for (a) **2**, (b) **3**, (c) **4** and (d) **5**.

We firstly explored the magnetic conduct of the isotropic gadolinium molecule, **1**, where some insight whether any interaction (i.e. ferromagnetic or antiferromagnetic) operates within the complex. The room temperature χ_M_*T* value (where χ_M_ is the molar magnetic susceptibility) for **1** is in good agreement with the expected values for two noninteracting Gd(III) ions 15.6 cm^3^ K mol^−1^ (where 15.8 cm^3^ K mol^−1^ is the expected value for two noninteracting Gd(III) ions with *g*_Gd_ = 2.00; *S* = 7/2). The χ_M_*T* stays practically constant along the whole temperature range, where a very small decrease at the lowest temperature occurs (leading to a value of 15.1 cm^3^ K mol^−1^) (Figure 2). This small downturn could correspond to antiferromagnetic exchange or dipolar interactions between the Gd(III)···Gd(III) ions (see below). Similarly, the anisotropic χ_M_*T*(*T*) behavior of the anisotropic analogues was also explored, yielding χ_M_*T* values close to the expected for the sum of noninteracting Ln(III) ions. The χ_M_*T* values of 23.4, 28.4, 28.2 and 24.6 cm^3^ K mol^−1^ were found for **2** to **5**, respectively, whilst expected values were 23.6, 28.3, 28.1 and 24.9 cm^3^ K mol^−1^ (for two: Tb(III), *g_J_* = 3/2, *J* = 6; Dy(III), *g_J_* = 4/3, *J* = 15/2; Ho(III), *g_J_* = 5/4, *J* = 8 or Er(III), *g_J_* = 6/5, *J* = 15/2). For **3** the χ_M_*T*(*T*) remains almost constant down to about 100 K, where it decreases reaching a value of 20.5 cm^3^ K mol^−1^ at 2 K (see Figure 3). A similar profile is observed for compounds **2**, **4** and **5**, where χ_M_*T*(*T*) of 19.11, 17.8 and 15.4 cm^3^ K mol^−1^ at the lowest temperature (i.e. 2 K) are correspondingly obtained. The decrease in χ_M_*T*(*T*) at low temperatures observed for all these compounds is due to a gradual depopulation of the Stark levels, i.e. depopulation of the crystal field split *m_J_* sublevels of the ground *J* multiplet, although magnetic exchange between the Ln(III) sites in these compounds could also cause such behavior (see Figure 3).

Molar magnetization (*M*_β_) as function of applied magnetic field at 2 K in the field range of 0–7 T was additionally investigated for all systems. The *M*_β_(*H*) for compound **1** shows a saturation value of 13.9 μ_B_, in very good agreement with the expected behavior obtained from the Brillouin function for noninteracting Gd(III) ions (Figure 2b). *M*_β_(*H*) measurements of complexes **2**–**5** at the maximum field (7 T) and the lowest temperature (2 K) yield values of 10.4, 11.0, 10.8 and 12.6 μ_B_, respectively (Figure 3 inset).

The very small downturn in the χ_M_*T* and the excellent agreement of the *M*_β_(*H*) data rules out any strong antiferromagnetic interaction operating within the gadolinium complex. The isotropic nature of compound **1** allows us to simulate the χ_M_*T* using the simple Heisenberg Hamiltonian, taking into account a single exchange interaction 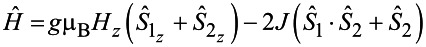. A small interaction is found sufficient to reproduce the small downturn in χ_M_*T*(*T*) (Figure 2a), giving *J*_Gd_ = −0.003(2) cm^−1^ between neighboring Gd(III) ions with a fixed *g*-value of *g* = 2.0. The calculation of the dipolar interaction [37] between the adjacent Gd(III) ions (i.e., Gd(1)···Gd(2)) at a distance of 6.8752(2) Å, leads to *D*^dip^ = −0.002 cm^−1^ (for a −2*J* Hamiltonian), demonstrating that the decrease in χ_M_*T*(*T*) is purely dipolar. A small zero field splitting of each individual Gd(III) could also be responsible for this behavior; however, due to the isotropic nature of Gd(III), this would be very small.

**Figure 4:**
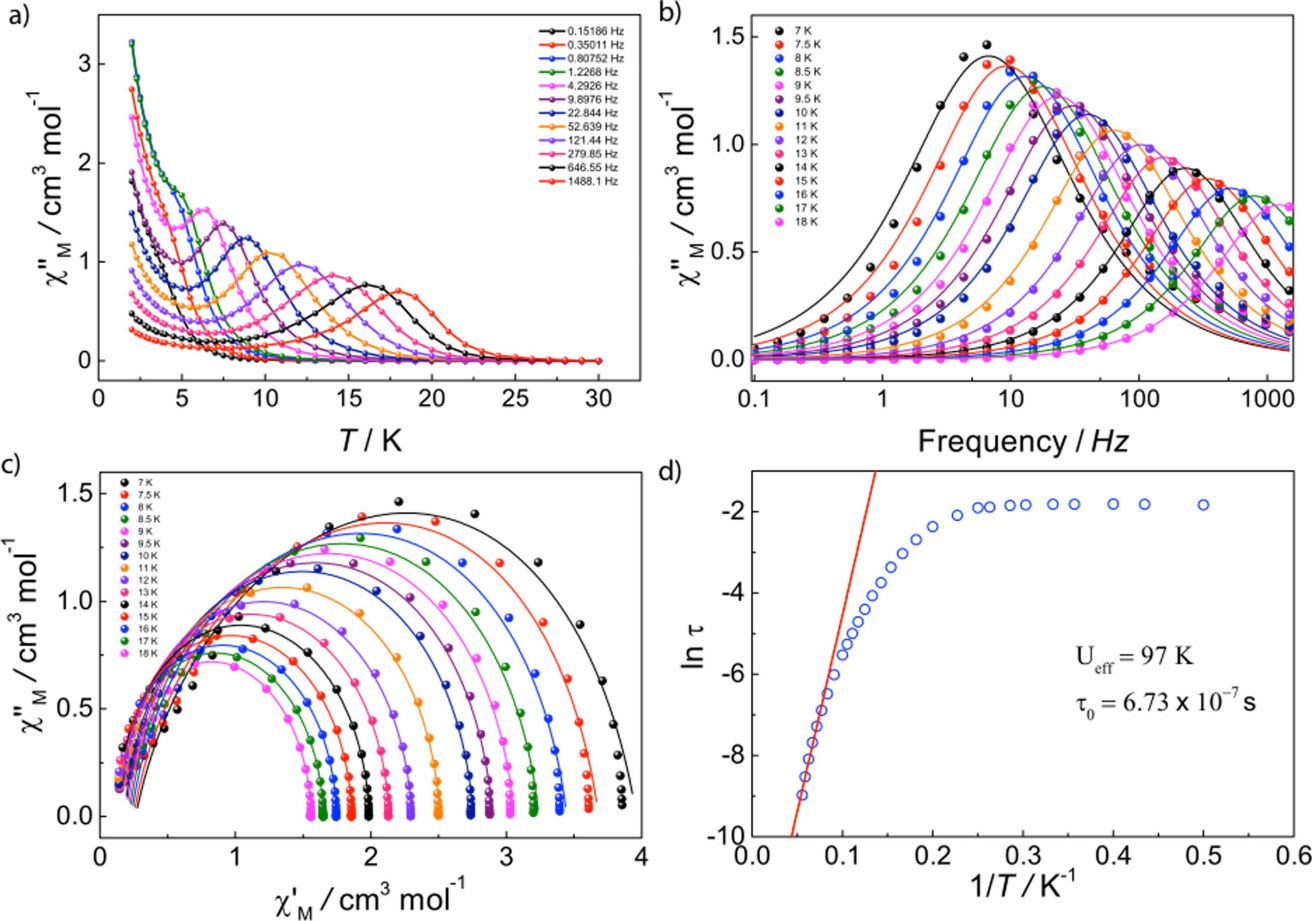
Experimental dynamic magnetic behavior for **3**. (a) χ"_M_(*T*) measured under a zero field (3.5 Oe AC field); (b) Experimental data and fits (solid lines) for χ"_M_(*ν*); (c) Cole–Cole plots (χ"_M_ vs χ'_M_) showing a single relaxation process above 4 K, where solid lines are fits of the AC susceptibility data to a modified Debye function; (d) Arrhenius treatment of χ"_M_ data for the high temperature process.

**Dynamic magnetic behavior.** To probe the presence of the slow relaxation of the magnetization for all anisotropic lanthanide analogues, the dynamic behavior for these was studied employing AC susceptibility measurements in the temperature range of 2–20 K and the frequency range of 0.1–1500 Hz with and without applied DC field. No SMM behavior was observed in compound **4** with or without an applied DC field. On the contrary, compound **3** shows a clear temperature dependent in-phase (χ'_M_(*T*)) and out of phase (χ"_M_(*T*)) component under zero field (Figure 4 and Supporting Information File 1, Figure S2). Likewise, compounds **2** and **5** show a frequency dependent behavior; however, no maximum is observed in either χ"_M_(*T*) or χ"_M_(*ν*), probably due to fast quantum tunneling (QT). The application of a small DC field is well known to suppress fast QT, which removes the zero-field degeneracy of the Kramers doublets at both sides of the barrier, allowing the observation of the dynamic magnetic behavior in the thermally activated regime [38–39]. We have therefore studied the optimal magnetic field (0–2 kOe), allowing the observation of the maximum in the frequency and temperature range for compounds **5** under a DC field of 800 Oe (Supporting Information File 1, Figure S3). Application of applied fields on compound **2** reveals the out-of-phase component; nevertheless, no maximum is achieved with either field in the given temperature and frequency range.

The out-of-phase (χ"_M_(*T*)) contribution of compound **3** reveals a single maximum at about 18 K for the highest frequency (*ν* = 1488 Hz), which shifts to lower temperatures with lower frequencies (Figure 4a). The peak becomes broader for lower frequencies where nonthermally activated relaxation pathways dominate. In the χ"_M_(*ν*), very small shifts of the maximum are present between 2 and 4 K, characteristic of the nonthermally activated pathways taking place. Above 4 K, the maximum shifts to higher frequencies with increasing temperature, indicating a purely, thermally activated regime (Figure 4b and Supporting Information File 1, Figure S2). Fitting the χ'_M_(*ν*) and χ"_M_(*ν*) to a single relaxation process in the temperature range using a Debye [40] model leads to magnetization relaxation times (τ) that were treated using the Arrhenius law, τ = τ_0_exp(*U*_eff_/*kT*), yielding an energy barrier (*U*_eff_) of 97 ± 3 K, where τ_0_ = (6.73 ± 0.4) × 10^−7^ s. The nonthermally activated regime is clearly marked in the Arrhenius plot at low temperatures range between 4 and 2 K, where the relaxation time is temperature independent with a tunneling frequency of 1.1 Hz, corresponding to a tunneling time τ_QTM_ of 0.145 ms. Cole–Cole fittings give a small distribution of relaxation times for an α-parameter of 0.02 < α < 0.2 (from 5 to 18 K), where a value of 0 would indicate no distribution (Figure 4c).

**Figure 5:**
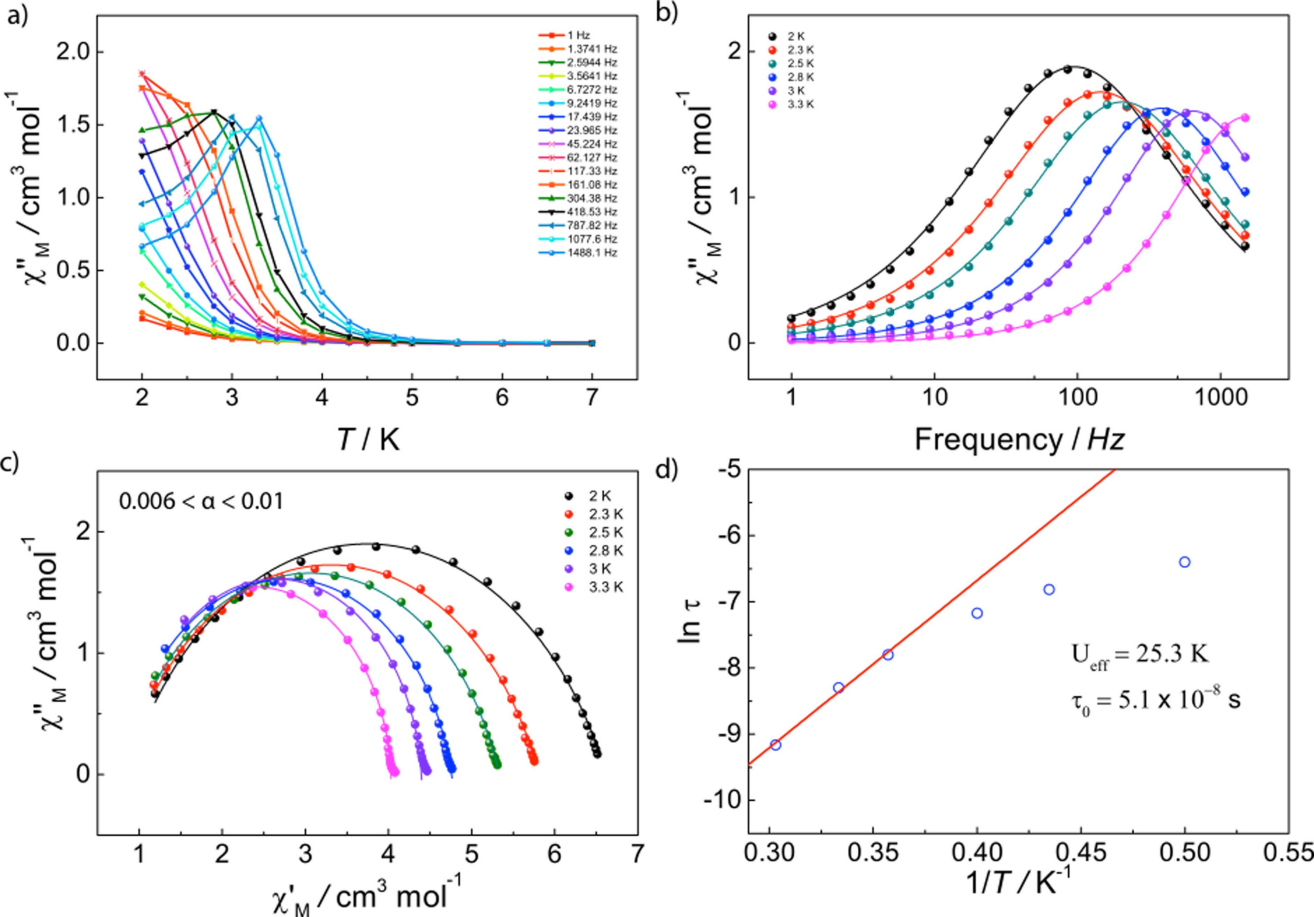
Experimental dynamic magnetic behavior for **5**. (a) χ"_M_(*T*) measured under an applied DC field of 800 Oe (3.5 Oe AC field); (b) Experimental data and fittings (solid lines) for χ"_M_(*ν*); (c) Cole–Cole plots (χ"_M_ vs χ'_M_) showing a single relaxation process, where solid lines are fits of the AC susceptibility data to a modified Debye function; (d) and Arrhenius treatment of χ"_M_ data for the high temperature process.

Compound **5** shows no SMM behavior at zero field; however, application of a small DC field lifts the degeneracy of the barrier, leading to the observation of the out-of-phase component. As evidenced by the clear frequency dependence of the signal (Figure 5a,b), compound **5** shows SMM features. A maximum at about 3.3 K (*ν* = 1488 Hz) is present in the χ"_M_(*T*), shifting to lower temperatures with frequency. The peak becomes broader at lower frequencies where nonthermally activated relaxation pathways dominate. A maximum is also shown in the χ"_M_(*ν*), shifting to higher frequencies with increasing temperature. We have similarly fitted both frequency dependent profiles, χ'_M_(*ν*) and χ"_M_(*ν*), to a single relaxation process in the temperature range employing a Debye model. The treatment of the relaxation times with the Arrhenius law gives an energy barrier (*U*_eff_) of 25 ± 2 K, τ_0_ = (5.11 ± 7) × 10^−8^ s, whilst Cole–Cole fittings render a very narrow distribution of α parameters of 0.006 < α < 0.01 (from 2 to 3.3 K) (Figure 5).

**Figure 6:**
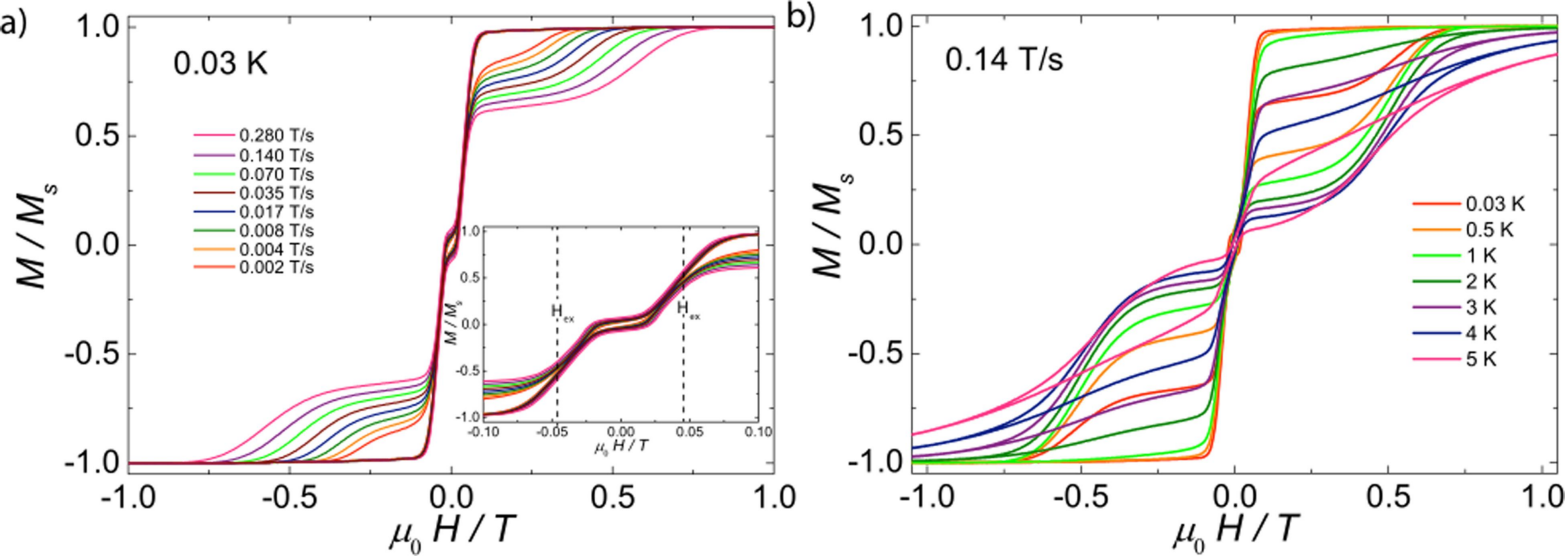
Single-crystal measurements of *M*(*H*)/*M_S_* vs µ_0_*H* measured on a micro-SQUID array for **3** (a) at 0.03 K at field sweep rates from 0.002–0.28 T s^−1^; inset: zoom view of the loops around zero field; and (b) at a sweep rate of 0.14 T s^−1^ at temperatures from 0.03–5 K.

**Magnetic studies at low temperature.** We have studied single crystals for complexes **2**, **3** and **5** at mK temperatures employing a micro-SQUID apparatus. No hysteresis loop was obtained for compounds **2** (Supporting Information File 1, Figure S5) and **5** (Supporting Information File 1, Figure S6) even down to 0.03 K. The magnetization of Er_2_ complex **5** has a sharp increase at μ_0_*H* = 0, indicative of a very fast tunneling rate of magnetization in this complex. This fact is in agreement with the AC magnetic susceptibility measurements discussed in the earlier section, where the fast tunneling can be slowed down as soon as a small field is applied. In contrast, well-resolved two-step hysteresis loops were obtained for complex **3** (Figure 6). The width of the hysteresis loop increases with decreasing temperature and increasing sweep rate, which is characteristic of SMM behavior. The loops are very typical for two antiferromagnetically coupled Ising-like spins. Around zero field, the loops have a S-shape with two sharp tunnel steps at positive and negative field, suggesting the presence of antiferromagnetic interactions between the two Dy(III) ions. At higher fields, around ±0.5 T, the loops have a broad step, which is strongly field-sweep-rate dependent and is due to a direct relaxation process between the ferromagnetic and antiferromagnetic spin states. The loops also show a small hysteresis at μ_0_*H* = 0, which comes from the fact that some of the molecules did not tunnel to the antiferromagnetic ground state, but remain in the ferromagnetic state. A similar observation has also been reported in an antiferromagnetically coupled Dy_2_ complex [41–42]. The mean exchange field (*H*_ex_) can be determined from the inflexion points at about ±0.046 T (inset of Figure 6), yielding the interaction constant *J* = −0.0019 cm^−1^ using *H*_ex_ = 2·*J*·*m_S_/g_J_*·μB, where *m_S_* = 15/2 and *g_J_* = 4/3. The strength of interactions between the two ions obtained from single-crystal measurement is comparable to that which resulted from the analysis of bulk magnetic data on the Gd compound **1**.

A good agreement with the dynamic behavior hysteresis loops were observed for compound **3**, confirming a better performing SMM compared to compound **5** (Supporting Information File 1, Figure S5). In the light of the observation of the hysteresis loops up to 5 K (Figure 6b), we also tested if hysteresis was also observable, employing a conventional SQUID magnetometer in the temperature range of 2–5 K. Hysteresis loops were indeed observed from 2–3.3 K in the range of ±0.3 T (Supporting Information File 1, Figure S4), confirming that **3** performs rather well as an SMM even at a very slow sweeping rate, i.e. 0.0003 T s^−1^.

**Figure 7:**
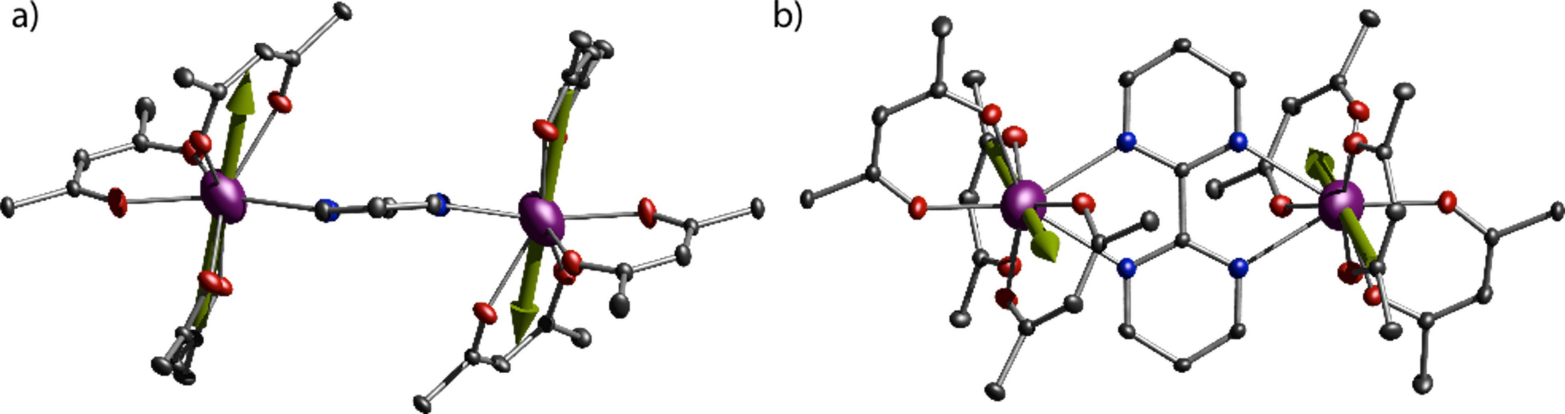
Magnetic axes obtained through the electrostatic method: (a) side and (b) top view for compound **3**. Color code: same as in Figure 1.

**Magnetic axes.** The dysprosium dimer compound, i.e. **3** exhibits a well-defined SMM behavior as evidenced in the χ"_M_(*T*), χ"_M_(*ν*) and hysteresis loops in the *M*_β_(*H*) results. This behavior has been associated with a well-defined *m_J_* = ±15/2 ground state doublet and highly axial *g*-tensors (ideally *g*_x_ = *g*_y_ = 0; *g*_z_ = 20) of the ^6^H_15/2_ manifold. To gain some insight into the orientation of the magnetic axes we employ an electrostatic method [43], which employs an electrostatic minimization of the 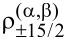 Sievers electron density [44] and a minimal valence bond model. The method gives the directionality of the magnetic axes for compound **3**, being collinear between the two Dy(III) sites, as expected from symmetric considerations (Figure 7) and almost perpendicularly aligned to the bipyrimidine plane. In such a case, the dipolar field between the two Dy(III) ions can easily be calculated [4] to be 0.03 T, which is less than 0.046 T observed in the micro-SQUID measurements. This means that the main contribution of the interactions between the ions is dipolar but some exchange coupling contributes as well. Undoubtedly, this co-parallel alignment is responsible for the SMM profile observed. Quantum tunneling is commonly accelerated by the noncollinear magnetic arrangement [45] and small exchange interactions [45–48], introducing further relaxation pathways. In compound **3** this is not the case, therefore the magnetic behavior could be associated with small exchange interactions, colinearity of the magnetic axis and a well-defined ground state of the individual Dy(III) ions.

### Scanning tunneling microscopy of [Tb(tmhd)_3_]_2_bpm

**Figure 8:**
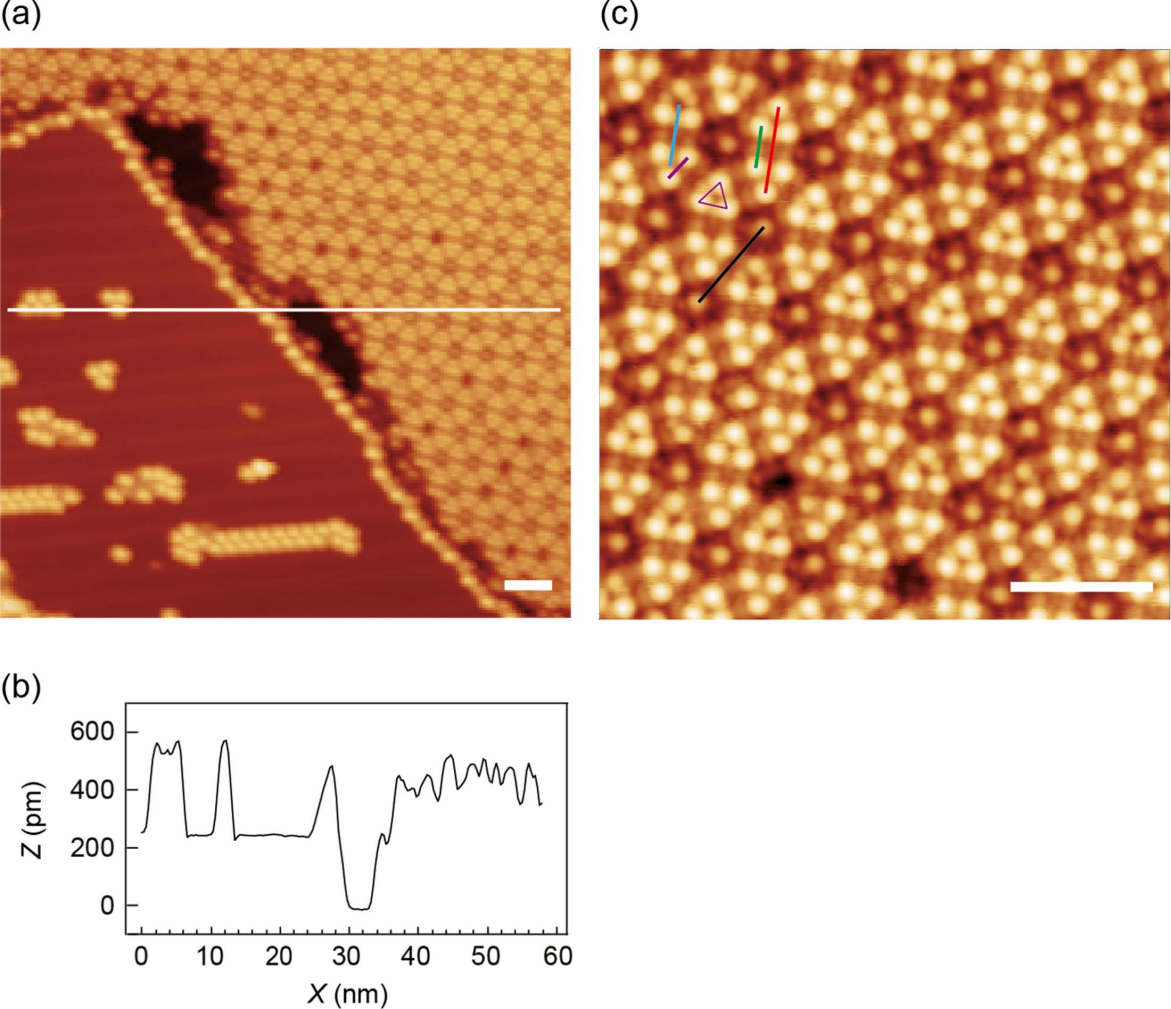
STM topography of **2** after deposition on Au(111). Image sizes are (a) 60 × 60 nm^2^ and (c) 20 × 20 nm^2^. White scale bars in the lower right corner in the images (a) and (c) indicate the length of 5 nm. (b) Cross section along the thin white line in (a). Set point: (a) 2.5 V, 10 pA, (c) 2 V, 40 pA. Length of the respective lines: black, 3.4 nm; red, 3.0 nm; green, 1.4 nm; blue, 2.0 nm; violet, 1.0 nm.

Scanning tunneling microscopy (STM) studies were performed at 5 K to investigate the structure and behavior of the compound at the single-molecule level. Several repetitions of the experiments suggested that the original molecular structure of **2** is substantially altered when deposited on the Au(111) surface. The experimental results of the deposition are summarized in Figure 8. There, the molecular phase forms a hexagonal surface pattern in a monolayer arrangement on the lower terrace of the Au(111) crystal plane (Figure 8a). Apart from the hexagonal molecular phase, small numbers of bright protrusions form a cluster-type arrangement on the upper terrace of the gold crystal (Figure 8b). The shape of these clusters as well as of the molecular phase differs substantially from the projected molecular structure, which can be derived from the single crystal measurement of the bulk compound (see Figure 1).

Obviously, some of the molecules are decomposed on the Au substrate, but the majority of the molecules seem to self-organize as trimolecular nodes. The cross section of the topography is shown in Figure 8b. The heights of the small clusters range from 350 to 450 pm. Heights of 400 pm and 280 pm have been reported on mononuclear ruthenium tris(β-diketonato) complexes on Ag(111) for two different conformations [49]. Thus, the height of the film (450 pm) is reasonable for the height of **2**, indicating a slightly distorted surface coordination in comparison to the literature. The small clusters in Figure 8a (left side) are likely decomposition products of [Tb(tmhd)_3_]_2_bpm (**2**), such as Tb(thmd)_3_, which may form at the gold surface. A magnified view of the molecular film is shown in Figure 8c. The pronounced hexagonal lattice of molecules is very regular and consists of triangular repetition. There, three bright protrusions form a triangle, which is highlighted in violet. The centers of the three dots lay in a distance range of about 1 nm, which corresponds well with the distance of the *tert*-butyl groups of the tmhd ligands between two adjacent molecules of the single crystal data. The average size of the nearly round bright protrusions is about 0.7 nm, which could describe the entire inner diameter of the Tb(tmhd)_3_ subunit within one dinuclear molecule. The inter-triangular distance from center to center (blue line) is 2 nm.

The underlying cause of the formation of the hexagonal structure remains unclear. However, size and shape analyses suggest that three molecules of **2** interlock with each other and form a piano stool subunit within the surface pattern. Further methodical investigation is currently underway. The STM study implies that the [Tb(tmhd)_3_]_2_bpm (**2**) molecule seems to be transferable onto the metal substrate with a certain amount of decomposition.

## Conclusion

The synthesis, molecular structure and magnetic properties of a series of five isostructural dilanthanide complexes **1–5** with a bipyrimidine bridging ligand were reported. The magnetic characteristics of all complexes were investigated by DC and AC SQUID-measurements, leading to the observation of SMM behavior in the Dy_2_- and Er_2_-containing systems, **3** and **5**. Clear out-of-phase components were observed for the Dy_2_-analogue **3** under zero field, whilst application of a small bias DC field to the Er_2_-analogue **5** slows down the quantum tunneling rate revealing the SMM properties. Furthermore, micro-SQUID studies show hysteresis loops for these complexes demonstrating that they retain their magnetization below a certain temperature, i.e. up to 5 K for the Dy_2_ complex **3**. To gain some insight into the molecular orientation of the magnetic axes of the two lanthanide ions, we have employed an electrostatic method, which gives a parallel alignment of the axes of the Dy(III) ions. These results demonstrate that SMM behavior can be achieved by linking two lanthanide metal ions (which exhibit single-ion magnetic anisotropy) with a 2,2’-bipyrimidine bridging ligand. The observed SMM character, with hysteresis loops observed as high as 5 K, make this class of bipyrimidine-bridged dilanthanide complexes promising systems to be sublimated onto surfaces. In this way, it is possible to study their magnetic behavior as single molecules or in thin film compositions. Due to its charge-neutral character, it was possible to sublime [Tb(tmhd)_3_]_2_bpm (**2**) onto a Au(111) surface. Preliminary results from scanning tunneling microscopy at 5 K suggest the formation of a homogeneous molecular film. Based on this result, it can be envisioned that the presented class of molecules, eventually equipped with linker-substituents, will function as active molecular entities that could be combined into spintronic hybrid device environments [14,50].

## Supporting Information

File 1 Additional experimental information.
