# Supplementary material for: Single-molecule magnet behavior in 2,2’-bipyrimidine-bridged dilanthanide complexes
Source: Beilstein J Nanotechnol. 2016 Jan 28;7:126–37. doi: 10.3762/bjnano.7.15 (PMC4734418; doi:10.3762/bjnano.7.15)
Supplement: File 1 — Additional experimental information. [file Beilstein_J_Nanotechnol-07-126-s001.pdf]

Supporting Information

for

Single-molecule magnet behavior in 2,2'-bipyrimidine-bridged dilanthanide complexes

Wen Yu[1*], Frank Schramm[1], Eufemio Moreno Pineda[1], Yanhua Lan[2], Olaf Fuhr[1], Jinjie Chen[3], Hironari Isshiki[3], Wolfgang Wernsdorfer[2], Wulf Wulfhekel[3], and Mario Ruben[1,4*]

Address: [1]Institute of Nanotechnology (INT), Karlsruhe Institute of Technology (KIT), Hermann-von-Helmholtz-Platz 1, D-76344 Eggenstein-Leopoldshafen, Germany, [2]Université Grenoble Alpes / CNRS, Institut NEEL, 25 rue des Martyrs, F-38000 Grenoble, France, [3]Physikalisches Institut (PI), Karlsruhe Institute of Technology (KIT), Wolfgang-Gaede-Str. 1, D-76131 Karlsruhe, Germany, and [4]Institut de Physique et Chimie des Matériaux de Strasbourg (IPCMS), CNRS-Université de Strasbourg, 23 rue du Loess, BP 43, F-67034 Strasbourg Cedex 2, France

Email: WenYu - wen.yu@kit.edu; Mario Ruben - mario.ruben@kit.edu

* Corresponding author

Additional Experimental Information

Contents

Table S1: Sublimation points, the chemical shift δ and IR spectra peaks of 1–5. 3
Table S2: Selected bond lengths [Å] and angles [º] for 1–5. 4
Table S3: Continuous shaped measures (CShM) for {Ln_2} obtained using SHAPE. 5

Figure S1: Views of the coordination polyhedra surrounding the lanthanide ions. Color code used: grey for bipyrimidine ligand, green, pink, orange and dark blue for the respective tmhd ligands........ 6
Figure S2: χ_M'(v), χ_M''(*v*) and χ_M'*T*(*v*) for 3 at zero DC field. Solid lines represent the fittings........ 7
Figure S3: χ_M''(*v*) for compound 5 at 2 K from 0 to 5 kG (solid lines are guide for the eye). 8
Figure S4: Hysteresis loops observed in the M_β as a function of applied magnetic field (*H*). 8
Figure S5: Magnetization vs. field on a single crystal of Tb_2 complex 2 at 0.03 K with different field sweep rates (left) and at a 0.07 T/s sweep rate in the 0.03-5 K temperature range........ 9
Figure S6: Magnetization vs field on a single crystal of Er_2 complex 5 at 0.03 K with different field sweep rates (left) and at a 0.14 T/s sweep rate in the 0.03–5 K temperature range........ 10

Table S1: Sublimation points, the chemical shift δ and IR spectra peaks of **1–5**.

Formula	Sublimation point / °C	^{1}H NMR (500 MHz, $CDCl_3$, 298 K) δ / ppm				IR (KBr disk) / cm^{-1}		
		H-1	H-2	Methin	*t*-Bu	ν(C=O)	ν(C=C)	ν(bpm)
**1** $[Gd(thmd)_3]_2bpm$	167 (1*10^{-3} mbar)	[a]	[a]	1.30	1.18	1576	1507	1420
**2** $[Tb(thmd)_3]_2bpm$	188 (6*10^{-2} mbar)	16.19 - 15.20	6.56 - 5.75	-0.74	1.20	1576	1507	1420
**3** $[Dy(thmd)_3]_2bpm$	165 (1*10^{-3} mbar)	20.03	6.00	-2.77	1.17	1576	1507	1421
**4** $[Ho(thmd)_3]_2bpm$	180 (2.5*10^{-2} mbar)		[b]			1576	1507	1424
**5** $[Er(thmd)_3]_2bpm$	160 (4*10^{-3} mbar)	40.32	27.48 - 23.37	-28.64 - -30.99	1.16	1577	1507	1425

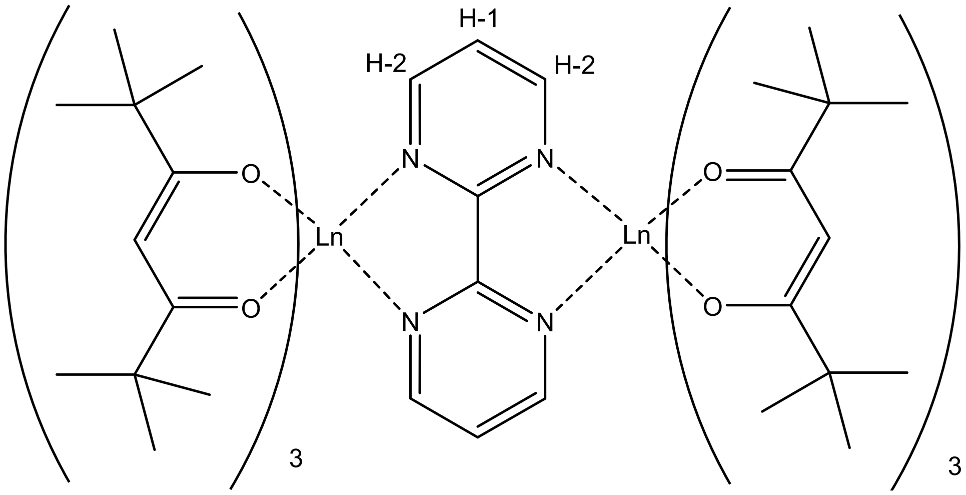

[a]These resonances are broadened beyond detection.
[b]The chemical shifts couldn't be detected.

Table S2: Selected bond lengths [Å] and angles [º] for **1–5**.

**1**	**Distance / Å**	**2**	**Distance / Å**	**3**	**Distance / Å**	**4**	**Distance / Å**	**5**	**Distance / Å**
Gd1-O1	2.363(3)	Tb1-O1	2.337(2)	Dy1-O1	2.330(2)	Ho1-O1	2.326(3)	Er1-O1	2.293(4)
Gd1-O2	2.318(3)	Tb1-O2	2.307(2)	Dy1-O2	2.294(2)	Ho1-O2	2.290(3)	Er1-O2	2.280(4)
Gd1-O3	2.277(3)	Tb1-O3	2.343(2)	Dy1-O3	2.296(2)	Ho1-O3	2.244(4)	Er1-O3	2.269(4)
Gd1-O4	2.322(3)	Tb1-O4	2.305(2)	Dy1-O4	2.260(2)	Ho1-O4	2.285(4)	Er1-O4	2.241(4)
Gd1-O5	2.321(3)	Tb1-O5	2.265(2)	Dy1-O5	2.293(2)	Ho1-O5	2.288(4)	Er1-O5	2.296(4)
Gd1-O6	2.352(3)	Tb1-O6	2.307(2)	Dy1-O6	2.324(2)	Ho1-O6	2.310(3)	Er1-O6	2.292(4)
Gd1-N1	2.631(4)	Tb1-N1	2.599(2)	Dy1-N1	2.581(2)	Ho1-N1	2.593(4)	Er1-N1	2.587(4)
Gd1-N3	2.613(4)	Tb1-N2	2.616(2)	Dy1-N2	2.604(2)	Ho1-N3	2.588(5)	Er1-N2	2.578(4)
Gd2-O7	2.344(3)					Ho2-O7	2.302(3)		
Gd2-O8	2.326(3)					Ho2-O8	2.288(3)		
Gd2-O9	2.285(3)					Ho2-O9	2.252(4)		
Gd2-O10	2.331(3)					Ho2-O10	2.301(4)		
Gd2-O11	2.321(3)					Ho2-O11	2.284(4)		
Gd2-O12	2.349(3)					Ho2-O12	2.308(3)		
Gd2-N2	2.631(4)					Ho2-N2	2.590(5)		
Gd2-N4	2.657(4)					Ho2-N4	2.618(4)		
**1**	**Angles / º**	**2**	**Angles / º**	**3**	**Angles / º**	**4**	**Angles / º**	**5**	**Angles / º**
O1-Gd1-O2	71.6(1)	O1-Tb1-O2	72.70(5)	O1-Dy1-O2	72.57(7)	O1-Ho1-O2	72.6(1)	O1-Er1-O2	73.2(1)
O3-Gd1-O4	73.0(1)	O3-Tb1-O4	72.20(5)	O3-Dy1-O4	74.13(7)	O3-Ho1-O4	73.9(1)	O3-Er1-O4	74.4(1)
O5-Gd1-O6	72.4(1)	O5-Tb1-O6	73.76(6)	O5-Dy1-O6	72.95(7)	O5-Ho1-O6	73.5(1)	O5-Er1-O6	72.9(1)
N1-Gd1-N3	61.7(1)	N1-Tb1-N2	62.07(5)	N1-Dy1-N2	62.37(7)	N1-Ho1-N3	62.5(1)	N1-Er1-N2	62.6(1)
O7-Gd2-O8	71.9(1)					O7-Ho2-O8	73.1(1)		
O9-Gd2-O10	73.2(1)					O9-Ho2-O10	73.3(1)		
O11-Gd2-O12	72.2(1)					O11-Ho2-O12	73.4(1)		
N2-Gd2-N4	61.0(1)					N2-Ho2-N4	62.2(1)		

Table S3: Continuous shaped measures (CShM) for $\{Ln_2\}$ obtained using SHAPE.

CShM	Gd(1)	Gd(2)	Tb(1)	Tb(2)	Dy(1)	Dy(2)	Ho(1)	Ho(2)	Er(1)	Er(2)
**OP-8**	35.832	30.374	30.219	30.219	30.205	30.205	30.263	30.151	29.855	46.201
**HPY-8**	23.057	22.242	22.339	22.339	22.442	22.442	22.328	22.549	22.330	36.352
**HBPY-8**	21.536	14.987	15.442	15.442	15.407	15.407	15.442	14.886	15.998	33.333
**CU-8**	15.995	8.231	8.851	8.851	8.796	8.796	8.460	8.218	9.076	31.299
**SAPR-8**	8.338	0.741	0.621	0.621	0.605	0.605	0.671	0.664	0.534	25.852
**TDD-8**	9.822	2.341	2.407	2.407	2.375	2.375	2.180	2.361	2.331	27.145
**JGBF-8**	19.999	15.997	16.085	16.085	16.138	16.138	16.572	15.786	16.555	33.117
**JETBPY-8**	22.099	27.493	27.490	27.490	27.633	27.633	28.002	27.740	28.138	39.774

OP-8 = (D_{8h}) Octagon
HPY-8 = (C_{7v}) Heptagonal pyramid
HBPY-8 = (D_{6h}) Hexagonal bipyramid
CU-8 = (O_h) Cube
SAPR-8 = (D_{4d}) Square antiprism
TDD-8 = (D_{2d}) Triangular dodecahedron
JGBF-8 = (D_{2d}) Johnson gyrobifastigium J26
JETBPY-8 = (D_{3h}) Johnson elongated triangular bipyramid J14
JBTPR-8 = (C_{2v}) Biaugmented trigonal prism J50
BTPR-8 = (C_{2v}) Biaugmented trigonal pris
JSD-8 = (D_{2d}) Snub diphenoid J84
TT-8 = (T_d) Triakis tetrahedron
ETBPY-8 = (D_{3h}) Elongated trigonal bipyramid

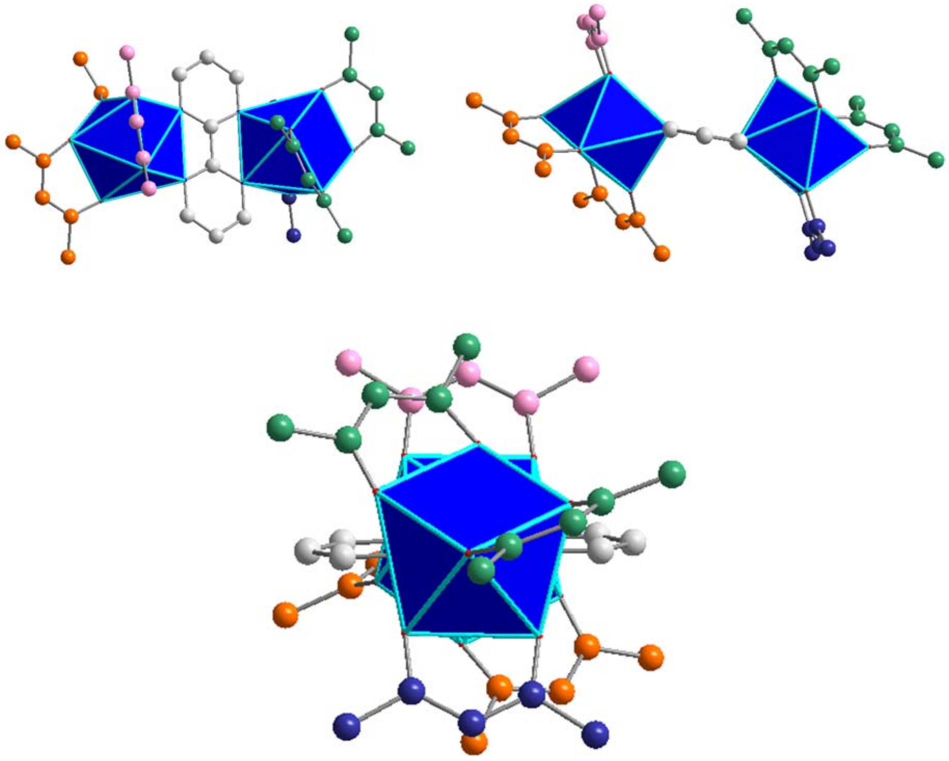

Figure S1: Views of the coordination polyhedra surrounding the lanthanide ions**.** Color code used: grey for bipyrimidine ligand, green, pink, orange and dark blue for the respective tmhd ligands.

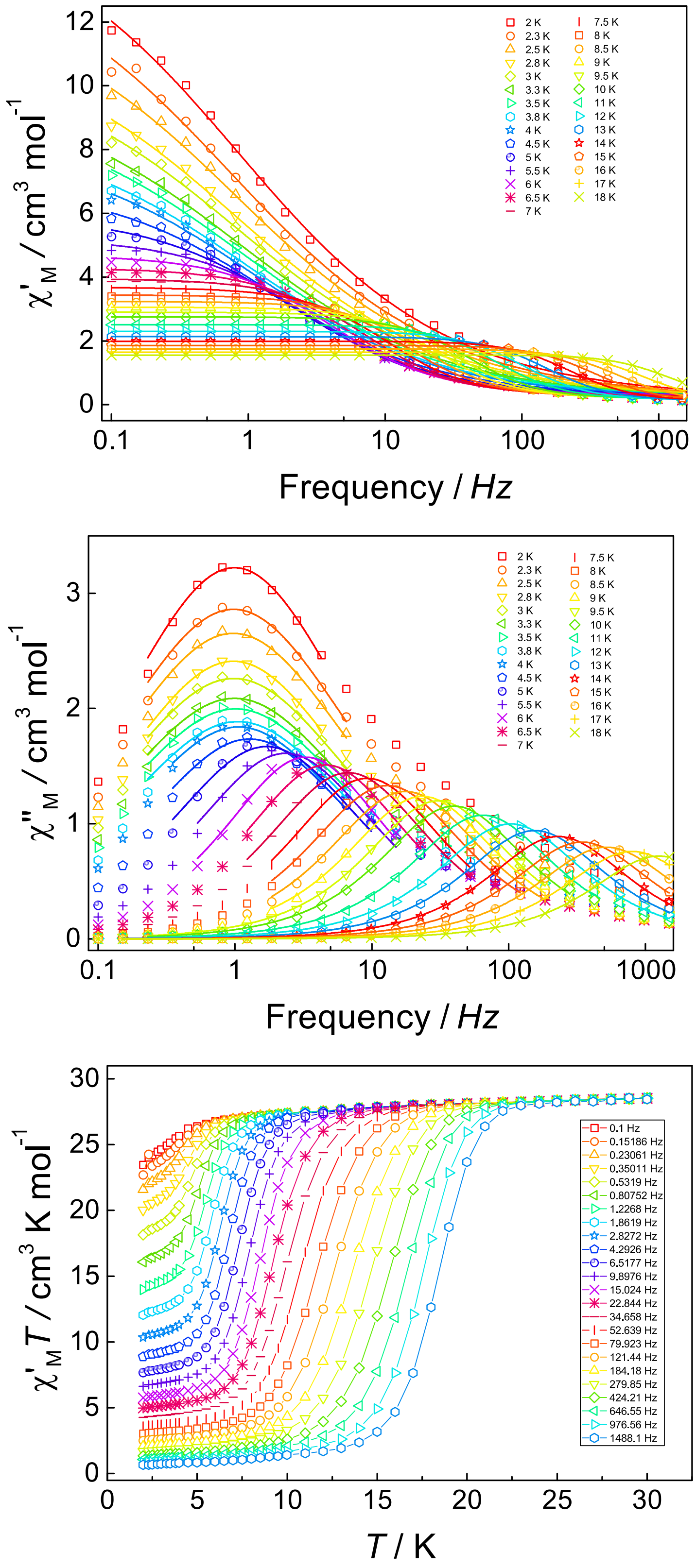

Figure S2: $\chi_M'(v)$, $\chi_M''(v)$ and $\chi_M'T(v)$ for **3** at zero DC field. Solid lines represent the fittings.

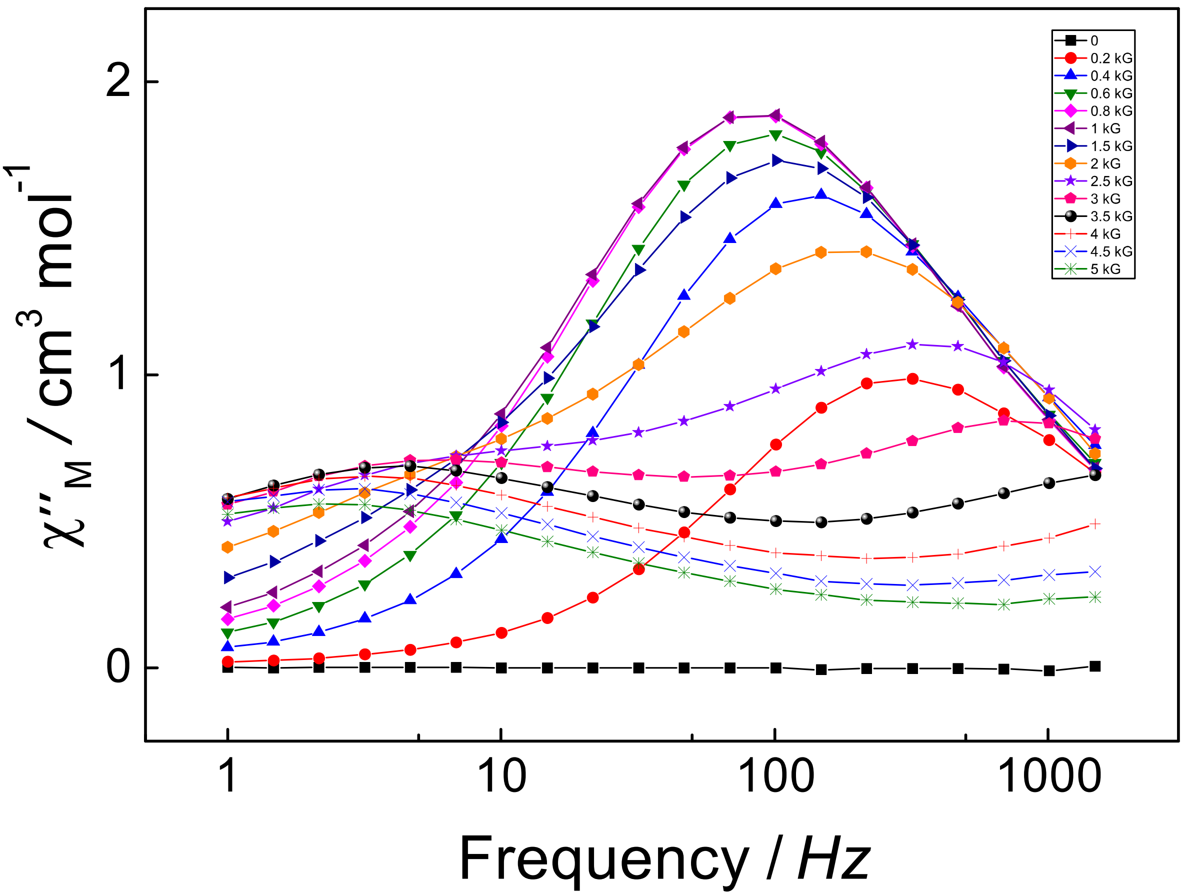

Figure S3: $\chi_M''(v)$ for compound 5 at 2 K from 0 to 5 kG (solid lines are guide for the eye).

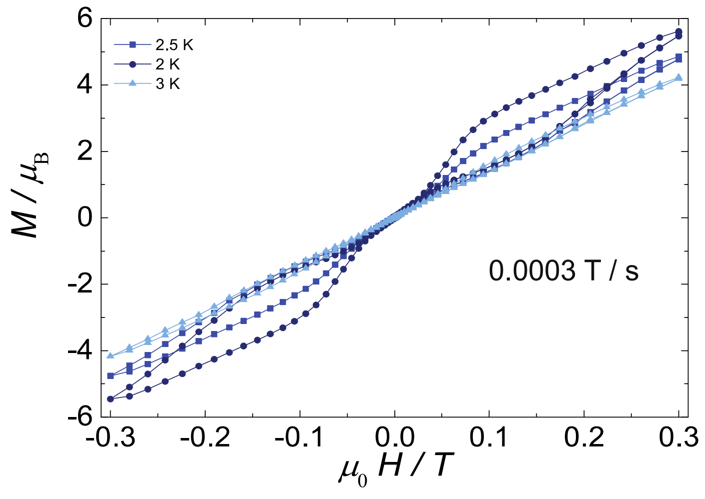

Figure S4: Hysteresis loops observed in the M_β as a function of applied magnetic field (H).

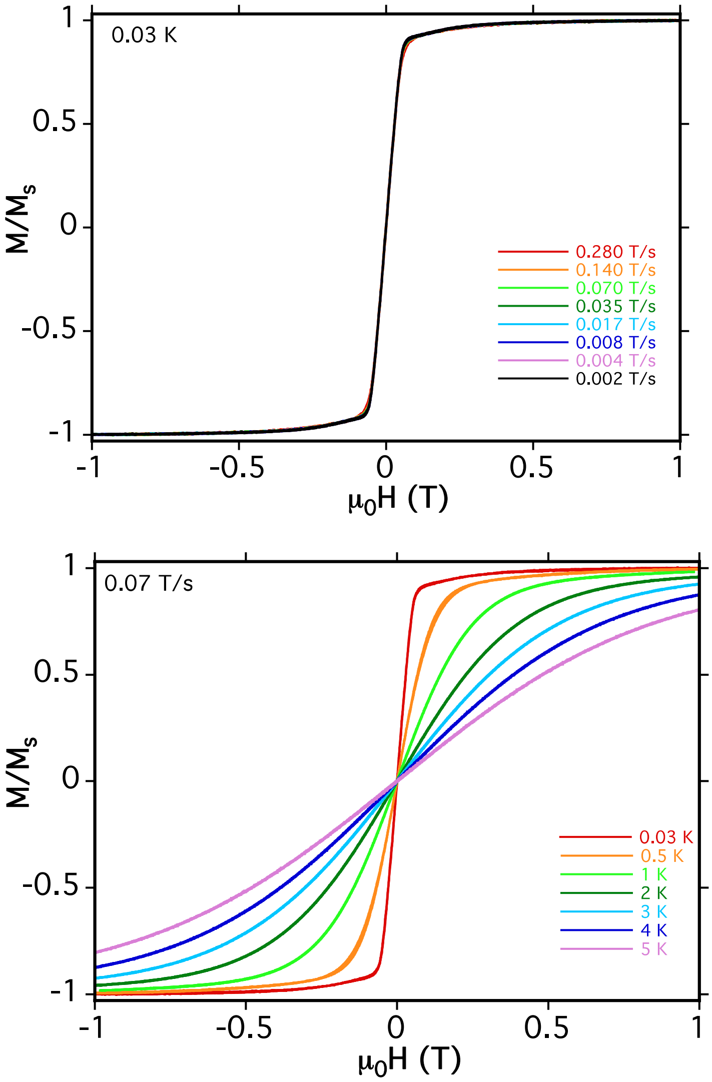

Figure S5: Magnetization vs. field on a single crystal of Tb_2 complex 2 at 0.03 K with different field sweep rates (left) and at a 0.07 T/s sweep rate in the 0.03-5 K temperature range.

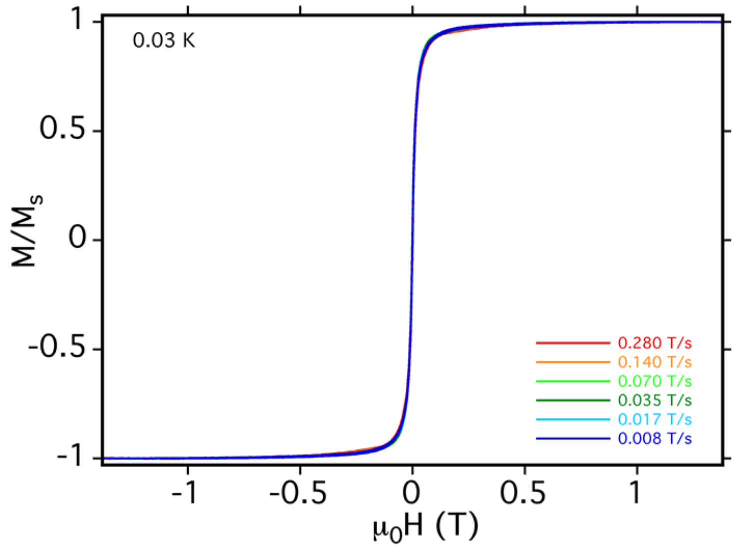

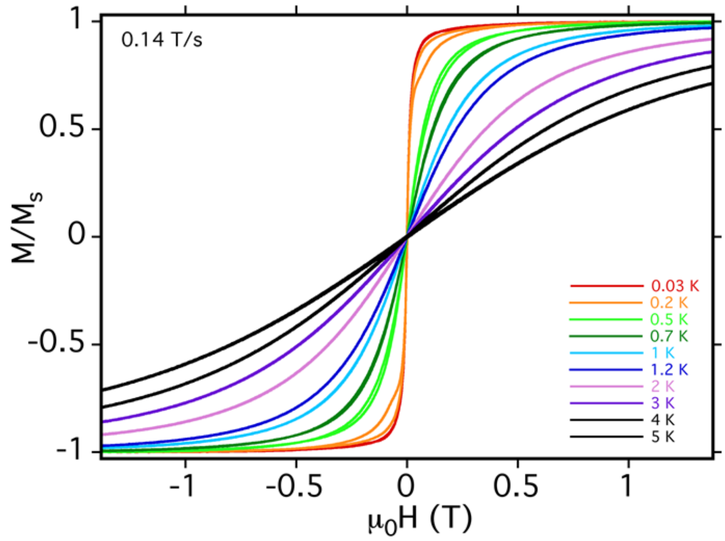

Figure S6: Magnetization vs field on a single crystal of Er_2 complex 5 at 0.03 K with different field sweep rates (left) and at a 0.14 T/s sweep rate in the 0.03–5 K temperature range.
